# Underwater Sensor Networks: A New Energy Efficient and Robust Architecture

**DOI:** 10.3390/s120100704

**Published:** 2012-01-10

**Authors:** Salvador Climent, Juan Vicente Capella, Nirvana Meratnia, Juan José Serrano

**Affiliations:** 1 Institut ITACA, Universitat Politècnica de València, Edifici 8G, València 46022, Spain; E-Mails: jcapella@disca.upv.es (J.V.C.); jserrano@itaca.upv.es (J.J.S.); 2 Pervasive Systems, University of Twente, P.O. Box 217, Enschede 7500 AE, The Netherlands; E-Mail: n.meratnia@utwente.nl

**Keywords:** underwater sensor networks, energy efficiency, fault tolerance, routing, scheduling and retransmission

## Abstract

The specific characteristics of underwater environments introduce new challenges for networking protocols. In this paper, a specialized architecture for underwater sensor networks (UWSNs) is proposed and evaluated. Experiments are conducted in order to analyze the suitability of this protocol for the subaquatic transmission medium. Moreover, different scheduling techniques are applied to the architecture in order to study their performance. In addition, given the harsh conditions of the underwater medium, different retransmission methods are combined with the scheduling techniques. Finally, simulation results illustrate the performance achievements of the proposed protocol in end-to-end delay, packet delivery ratio and energy consumption, showing that this protocol can be very suitable for the underwater medium.

## 1. Introduction

Underwater Wireless Sensor Networks (UWSN) and terrestrial Wireless Sensor Networks (WSN) share common properties but they have many differences too. These differences necessitate specialized new protocols for underwater communication.

Cost of the nodes is one of the differences, as underwater wireless sensors are expensive partially because of their more complex transceivers. Another difference relates to the deployment costs. Deployment of underwater sensors, especially in deep waters, can be difficult and expensive. Therefore, an UWSN has to be carefully studied and planed (in terms of performance evaluations, simulations, and tests) before its deployment. Power is another important difference since underwater communications requires higher power than the terrestrial network. But, the most important difference is the communication medium. Underwater communications cannot use Radio Frequency (RF) signals since they have an enormous attenuation in the subaquatic medium. Therefore, acoustic signals are used underwater.

Acoustic waves present different signal attenuations depending on distance and frequency [1]. In addition, the signal spreading is proportional to the distance due to the expansion of the wave-fronts and can be in a cylindrical or spherical form [2]. Another problem faced by underwater communication comes with the signal propagation, which is 1,500 m/s and five orders of magnitude lower than in RF. In radio-frequency networks this delay is negligible but in underwater acoustic networks it is important to be considered.

Given this long propagation delay, the original medium access techniques developed for RF networks, such as TDMA or CSMA, do not perform well underwater [3]. TDMA may need large time-guards to deal with the long propagation delay and the hidden terminal problem is amplified in CSMA networks. The use of a scheduling algorithm to organize the transmissions can, on one hand, avoid collisions, and on the other, reduce or even remove the time-guards, taking advantage of the propagation delay and overlapping transmissions.

On the other hand, new routing protocols that meet these requirements and offer high energy efficiency are needed. It would also be desirable to submit additional features such as robustness and fault tolerance, which are not currently provided in the proposals made in the literature.

In this paper, we propose EDETA (Energy-efficient aDaptive hiErarchical and robusT Architecture) as a routing protocol for UWSN, which was recently adapted to this new transmission medium [4]. Moreover, we aim to analyze impacts of different delay-aware and non-delay-aware scheduling and retransmission techniques when applied underwater, in order to optimize the protocol performance in terms of energy consumption, packet delay, number of duplicate packets, and packet loss using simulations in Network Simulator 3 [5]. In addition the paper presents a comparison between the protocols EDETA-e and DBR [6].

The remaining of this paper is organized as follows. In Section 2, state-of-the-art underwater MAC and routing protocols as well as scheduling algorithms will be presented. Section 3 provides a description of EDETA, while Section 4 introduces different scheduling and retransmission techniques used in the experiments, and describes our simulation, the comparison between EDETA-e *vs.* DBR and the obtained results. Finally, in Section 5 conclusions are drawn and our future work is highlighted.

## 2. Related Work

Underwater acoustic transmission has been heavily studied during the last decade. Recently, significant advances in MAC and routing protocols for underwater sensor networks have been achieved. Good surveys reviewing the recent advances and challenges in underwater sensor networks can be found in [7–9].

### 2.1. MAC Protocols and Scheduling Algorithms for UWSN

The propagation delay is one of the most studied factors in underwater MAC protocols. Researchers have been trying to adapt existing protocols and have been proposing new ones in order to address the differences between terrestrial and underwater acoustic networks.

The original FAMA (Floor Acquisition Multiple Access) protocol [10] prevents packet collisions provided that the RTS and CTS frames are long enough. Given the long propagation delay of the underwater acoustic medium, these packet lengths are very high, hence Molins *et al*. propose in [11] the Slotted FAMA MAC protocol. This protocol provides some energy savings since nodes do not have to transmit long RTS/CTS frames. However in this case, the slot length needs to be equal to the maximum propagation delay plus the transmission time of a CTS packet, which can lead to a low channel utilization.

T-Lohi (Tone-Lohi), a hybrid between RTS/CTS and CSMA, is proposed in [12]. This protocol adapts automatically the contention time to the number of contending nodes. The nodes send a short packet called tone prior to the actual data packet to count the number of terminals contending for the channel. If a node does not receive any other tones, it starts the transmission. However, if it receives more tones, it adapts its backoff time depending on the number of tones received. The channel utilization of this protocol is within 30% of the theoretical maximum.

Pompili *et al*. advocate for a CDMA-based MAC [13]. Their proposed MAC switches at each sender node from an ALOHA scheme, to transmit the header, to a CDMA-based scheme, to transmit the payload. This payload and the header are sent back-to-back in one transmission. A node first sends a short header with the spreading code and sends immediately the payload using this spreading code. Results show that this MAC scheme can achieve high network throughput in deep water communication and can dynamically adapt to compensate the multipath effect in shallow waters.

There are some proposals that take advantage of the propagation delay and overlap multiple transmissions in order to increase the throughput. Authors in [14] propose ST-MAC, which is a centralized scheduling algorithm that constructs a Spatial-Temporal Conflict Graph (ST-CG) to describe the conflict delays. Authors take a vertex colouring approach and model the graph by assigning a color to each of its edges. In order to solve the vertex-colouring problem, ST-MAC divides the transmissions into multiple timeslots which may lead to suboptimal results [15].

STUMP-WR [16] is also able of overlapping different transmissions using TDMA slots and scheduling them in a distributed manner. Given the distributed nature of the protocol, nodes can only use local information to perform their schedule, which may lead to suboptimal schedules. Moreover, it may not avoid all the possible interferences when facing the hidden terminal problem.

In [15], van Kleunen *et al*. introduce a set of scheduling constrains to avoid interferences and propose a centralized scheduling algorithm to take advantage of the propagation delay. Later in [17] they extend this set of scheduling constrains to allow the schedule to be performed in a distributed manner using clusters of nodes.

### 2.2. Routing Protocols for UWSN

As mentioned before, one of the main characteristics of the underwater acoustic channel is the propagation delay. Since it is five orders of magnitude higher than its radio frequency counterpart, different routing protocols have been proposed to mitigate this effect.

A centralized routing protocol is proposed in [18] to mitigate the high packet delay using a sliding window approach. The main drawback of this protocol is the increasing need of different transmission channels with the number of nodes.

Zorzi and Casari study in [19] the effects of the differences between the terrestrial and underwater transmission mediums and the relationship between energy consumption and different radio modes. Furthermore, they design a set of new routing protocols considering these studied factors. Although they make the assumption that each node has information about its position, it is not specified how localization is performed.

There are some approaches that use geographic routing protocols. In the VBF (Vector-Based Forwarding) algorithm [20] a node forwards a packet if the node is close enough to the estimated routing vector. The sender encapsulates into the data packet its position and the receiver position. With this information an intermediate node will forward the packet if it is close enough to the routing path.

DBR (Depth Based routing) [6] routes the messages from the bottom of the ocean to the surface using only depth information. The depth of the source node and the depth of the forwarder are attached to the packet, that way, an intermediate node forwards a packet only if its depth difference with the forwarder is higher than a certain predefined threshold. In addition, an intermediate node has to wait to forward a packet for a certain amount of time called the holding time during which, if a copy of the packet is received, the transmission is cancelled. This way, the protocol avoids flooding and the routing can be made without exact location information.

A similar algorithm to DBR is DSR (Direction-Sensitive Routing) [21]. This algorithm utilizes a fuzzy logic inference system to decide which nodes should forward a packet based on the distance and angle between two neighbouring sensor nodes, and the remaining energy left in the sensor node. In addition, in order to save energy, the protocol restricts the forwarding of the packet when the broadcast tree grows larger than a specified tree level. According to the authors, DSR can achieve the same packet delivery ratio as DBR but with lower end-to-end delay and less energy consumption.

QELAR (Q-learning-based Routing) is presented in [22]. It is an adaptive routing protocol based on a machine learning approach. When a node needs to transmit data, it piggybacks some state information. Every time a node receives a packet, even if it is not its destination, it reads the added state information and updates its state and routing function. Authors compare performance of QELAR *versus* the VBF algorithm and conclude that QELAR can achieve the same performance in terms of delivery rate and routing efficiency, but with more energy efficiency.

In [23] a centralized routing protocol based on geographic information is proposed. The master node computes the topology and the routing paths. The main drawback of this protocol lies in the complexity of the algorithm that computes the topology and the routing paths, since it is NP-complete.

Minimum Cost Clustering Protocol (MCCP) is a distributed clustering protocol proposed in [24]. The authors propose a cluster-centric cost-based optimization problem for the cluster formation. Although cluster-heads have the ability to send the data in a multi-hop manner to reach the sink, all nodes are supposed to be able to reach the sink. The cluster-head selection algorithm does not assure that the cluster-heads far away from the sink are able to relay their data to other cluster-heads.

In [25] authors present MPR (Multi-Path routing). This routing algorithm tries to minimize the end-to-end delay in underwater networks by sending the same packet through different routes in a 2-hops neighbourhood using relay nodes. This algorithm achieves lower end-to-end delay than VBF with a better packet delivery ratio.

## 3. Energy-Efficient aDaptive hiErarchical and RobusT Architecture

EDETA (Energy-efficient aDaptive hiErarchical and robusT Architecture) is a routing protocol originally proposed for WSN [26,27] and recently adapted to UWSN [4]. It is a hierarchical protocol and nodes arrange themselves in clusters with one of them acting as a cluster-head (CH). The CHs form a tree structure between themselves in order to send the collected and aggregated data from the other nodes to the sink in a multi-hop manner. The protocol supports more than one sink in order to provide more scalability and some fault tolerant mechanisms.

Operation of the EDETA protocol is divided into two phases, (i) the initialization phase and (ii) the normal operation phase. During the initialization phase, clustering is done and cluster-heads are elected. During the normal operation phase, the nodes send their data periodically, at their scheduled times, to their CHs. Finally, cluster-heads send their data to their parents until the data reaches the sink.

In this protocol, similarly to the LEACH election mechanism [28], the CH election mechanism is distributed among the nodes in the network. Moreover, in order to distribute the energy consumption, the network structure is broken up after a certain amount of time and the initialization phase starts again. An enhanced version of EDETA, called EDETA-e (EDETA-enhanced), also allows the designers of the network to accurately plan and choose nodes acting as CHs. In this variant of the protocol the initialization phase is done only once.

The CH election is based on a random number that must be lower than a calculated threshold, *T*(*n*), calculated by Equation (1):

(1) $$T(n)=\frac{c}{|N|-2c}\times \alpha ,n\in N$$

where *c* is desirable number of clusters in the network, *N* is the set of nodes in the network and *α* is a parameter whose value will depend on the time at which the equation is computed.

The nodes will compute the above equation in two cases: (i) at the beginning of the network configuration, in this case *α* = 1; and (ii) during the network configuration, if there is any CH that needs some normal nodes to become CH. In the latter case *α* has to be greater than 1 in order to increase the probability of becoming CH.

If the number randomly generated is lower than the calculated threshold, a node can become a CH only if its remaining energy is greater than value calculated by Equation (2):

(2) $$E(n)=E_{T}\times \frac{2T_{\mathrm{Config}}}{2T_{\mathrm{Config}}+\mathrm{MA}X_{\mathrm{Rounds}}T_{\mathrm{SuperFrame}}},n\in N$$

where *E_T_* is the average of the remaining energy of the CHs around the node and *T_Config_*, *T_SuperFrame_* and *MAX_Rounds_* are protocol parameters that will be discussed later in Section 3.1.

EDETA is a time-constrained protocol. As shown in the next section, the different phases of the protocol are time constrained. This way, EDETA can be used in applications in which time is an important variable.

### 3.1. Operation

As stated before, EDETA’s operation is divided into two phases, (i) the initialization phase and (ii) the normal operation phase. There are two variables that limit the duration of these phases. The *T_Config_* variable limits the initialization phase and the *T_SuperFrame_* variable limits one round of the normal operation phase. The normal operation phase has a limited number of rounds defined by the parameter *MAX_Rounds_*. Hence, the normal operation phase lasts for *MAX_Rounds_* *× T_SuperFrame_*.

**Figure 1.**
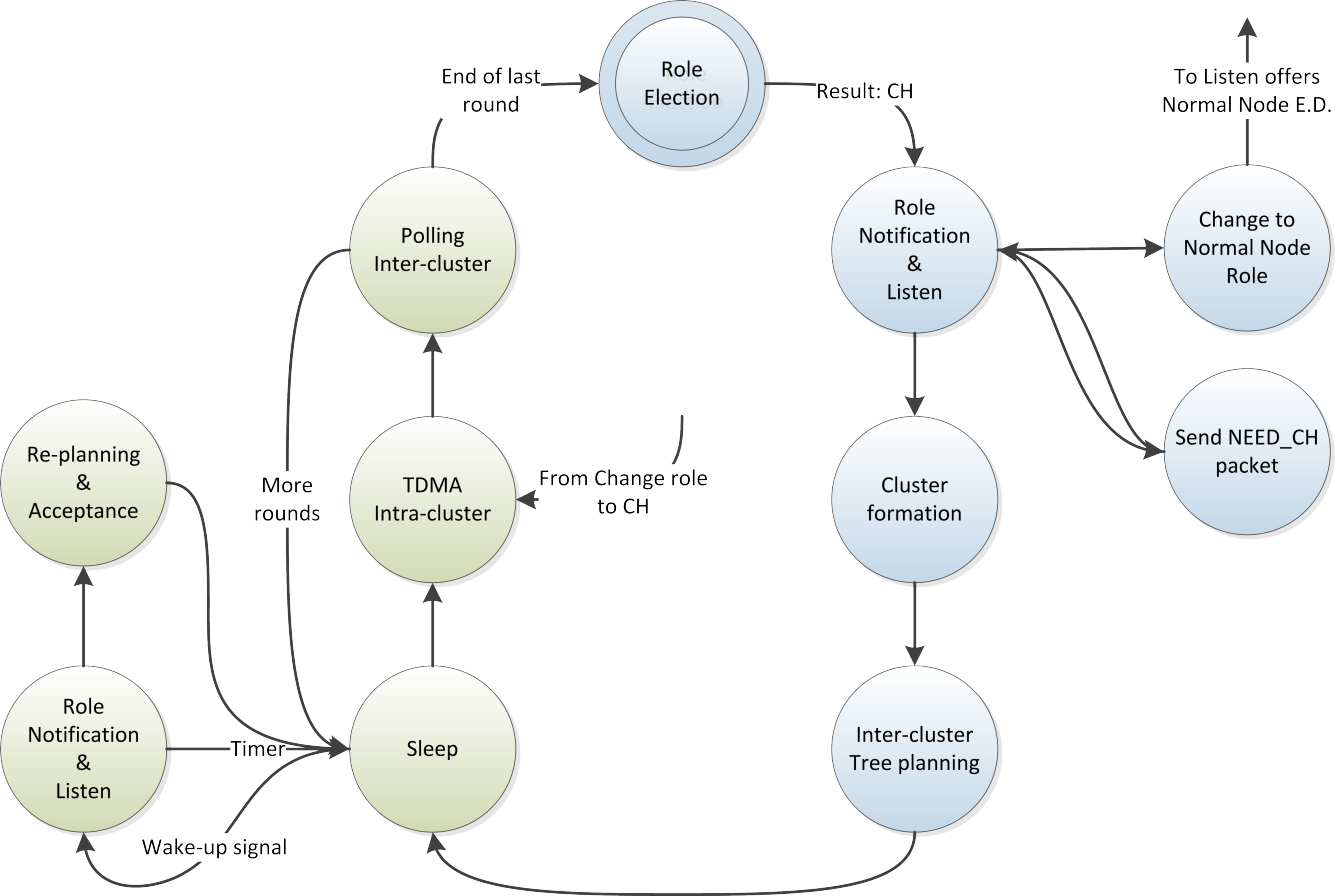
State Diagram of a CH.

**Figure 2.**
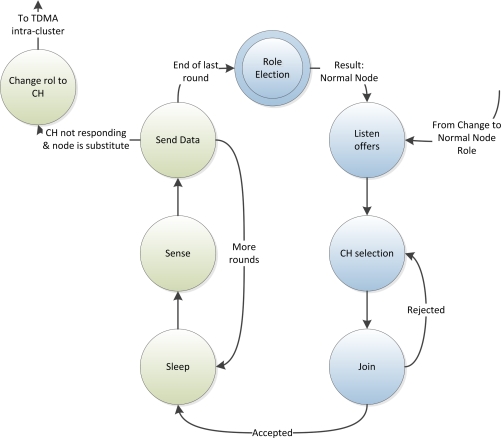
State Diagram of a Normal Node.

Figures 1 and 2 depict the state diagrams for the CHs and normal nodes respectively. The blue states are the ones corresponding to the initialization phase and the green states correspond to the normal operation phase.

All nodes start at the Role Election state, after which, each node starts listening for announcement of CHs and, if it becomes a CH, it starts announcing itself. Then, the network structure and network schedule is done distributedly and autonomously by the nodes. After that, the initialization phase finishes and the nodes start the normal operation phase, which lasts a certain amount of time as defined above. In the following sections, the initialization and normal operation phases are explained in detail.

#### 3.1.1. Initialization Phase

In this phase the network structure is built, normal nodes join their elected clusters and these clusters form a tree to deliver the data to the sink. This phase is divided in three sub-phases.

In the first part, with duration of a half *T_Config_*, each node decides on its own if it is going to be a CH, based on the procedure explained before.

When a node decides to be a CH it sends HEAD messages to announce its role to the rest of the network. At the same time a CH starts receiving HEAD messages from the others, it decides which CH is going to join in order to send its data to the sink. This decision is based on the signal strength of the HEAD messages. But a CH will only try to join another CH if the last one has established a path to the sink. That is, if it can communicate with CHs that can reach the sink directly or through others CHs.

Meanwhile, nodes which have decided to be leaf nodes start receiving HEAD messages too. They store them to decide to which CH they might join in the second part of this phase. The selection of the CH for these nodes follows the same criteria as selection of parent CH.

If a CH does not receive any HEAD message, it sends a NEED_CH message. When a normal node receives it, it reruns the procedure, but with an increased value of *α*, to decide whether it is going to be a CH. When *α* is increased the probability of a node to become a CH increases too. This probability is increased because the network needs to be built as fast as possible in order to save energy. This mechanism along with the first random distribution of CHs allows the protocol to adapt rapidly the population of CHs to the needs of the network.

At the end of this sub-phase, the tree structure is built and normal nodes have the necessary information to decide to which clusters they are going to join.

In the second sub-phase, with duration of a half *T_Config_*, normal nodes start to join their selected clusters. In the response message, their corresponding CH sends the TDMA schedule in which each node has to send its data. After that, the normal nodes enter the low-power state.

A CH can only allow a limited number of nodes to join in. This number is given by *MAX_Soft_* and *MAX_Hard_*. A CH accepts all the join request petitions until it reaches its *MAX_Soft_*. After that, it will only accept join petitions that have activated a last resort bit. When a CH reaches the *MAX_Hard_* threshold, it will no longer allow new joins.

Finally, in the third sub-phase, with duration of one *T_Config_*, each leaf CH in the tree sends to its parent the amount of time needed to have all the data recollected from his nodes. The parent collects this information and decides the time schedule in which its children can send it the data. After that, the parent node repeats the process with its own parent, sending the amount of time needed to collect all the data from its nodes and its children. This process continues until the entire tree is scheduled.

#### 3.1.2. Normal Operation Phase

At this moment, the network structure is done and every node must send its data to the CH at the scheduled time. During the remaining time, the nodes enter in the low-power state. When a CH has received the data from all its nodes, it aggregates it and sends it to his parent at the established time.

As said before, the parent of a cluster informs in which time its children have to send their data. Children CHs will send their data at the scheduled timer or, optionally, they can wait to send the data until they receive a POLL message from their parent. This allows the parent to decide exactly when the data will be sent and makes applicable some fault tolerant mechanisms, without inquiring collision of messages. This optional mechanism will be further discussed in Section 3.2.

This phase is repeated during some rounds, with duration of *T_SuperFrame_*, defined by parameter *MAX_Rounds_*. After that, the network structure is considered obsolete and every node restarts from the beginning at the initialization phase.

### 3.2. Fault Tolerant Mechanisms

Given the fact that UWSNs are prone to errors and that they are even more erroneous than WSN due to the harsh environment, it is mandatory to provide the protocol with some fault tolerant mechanisms to allow it recover from different node malfunctioning.

One of the biggest problems of this protocol resides in a malfunction of a CH. If a CH falls down, all the nodes in the cluster will not be able to send their data. In addition, the CH below them in the tree will never reach the sink.

To reduce the impact of this kind of troubles, the substitute node role has been introduced. A substitute node is a node within the cluster that shares the schedule with the CH and takes its place if it falls down.

The substitute node is selected by the CH in the initialization phase, based on the energy of the node and the proximity between them. To allow normal nodes to be in a low power state as much as they can, the normal node will be notified in the first round of the normal operation phase. This way, if a CH falls down, its substitute node will detect it and will take its place acting as the CH.

Another possible problem resides on a sink fall down, for which there is no solution in single-sink networks. As previously mentioned, the protocol allows multiple sinks in the network. Each sink is the root of its CHs tree. If a sink falls down, its tree becomes root-less. In this case, the CHs in the root-less tree have to integrate within the other trees. In order to do so, the CHs at the first level of the tree (which directly communicated with the sink) will detect that the sink is down as messages are not answered. In that moment, they have to inform all the CHs about this fact waking them up, using a low-power radio triggered wake-up [29,30]. By sending a wake-up signal, all nodes in the network will wake up and the CHs within the root-less tree can send their association request to join the remaining trees.

There is one more fault tolerant mechanism to be exposed. When a node has a temporally malfunction and performs a reset (or even when a new node is deployed), it has to be reinserted in the network. As previously mentioned, if this node was a CH the network will continue to work thanks to the substitute node role.

As this node is starting from the beginning, it has to perform the role election mechanism. On one hand, if it becomes a normal node, it will start listening to the channel in order to learn the CHs around it and, when it realizes that the network activity is not the expected one, it has to use the wake-up signal to wake up its nearby CHs and to join them by the usual mechanism. On the other hand, if it becomes a CH it will start sending HEAD messages but no one will respond to them. So, it will use the wake-up signal explained for the normal nodes.

### 3.3. EDETA-Enhanced

EDETA-e is a version of EDETA protocol that enables control over the network formation and over the delays. With EDETA-e, the engineer and not the protocol decides which node becomes a CH. This way, the engineer chooses where to place CH nodes and which type of power supply they will have.

Since CHs are fixed at design time, EDETA-e only considers one initialization phase. After that, all nodes will always remain in the normal operation phase.

In underwater acoustic networks, where current state-of-the-art transceivers have more power consumption compared with their radio-frequency counterpart and the propagation speed might vary over time, having a planed and fixed infrastructure can help the deployment of underwater networks. Moreover, since GPS signals are heavily attenuated underwater, the localization of sensor nodes and AUVs (Autonomous Underwater Vehicles) is not trivial and the fixed nodes can be used for localization proposes.

## 4. Simulations

In this section, EDETA protocol will be evaluated simulating its enhanced version. First of all, in Section 4.1 different tests are conducted in order to study the suitability of the protocol for the underwater medium.

Since the underwater medium is a harsh environment, in Section 4.2 different retransmission techniques for the EDETA-e protocol are evaluated along with different scheduling mechanisms. The reason behind doing so is to identify which technique is more efficient and convenient for each situation.

Finally, in Section 4.3 a comparison of the EDETA-e protocol is made with the DBR protocol in terms of packet delay, energy consumption and packet delivery ratio.

Unless stated differently, all nodes except CHs and sink start with 150 Joules of energy. The transmission power was set to 0.203 wats, the reception and idle power is set to 0.024 wats and the sleep power to 3 *×* 10^−6^ watts. The transmission range of the nodes is limited to 100 m adjusting the model transmission power. These values were extracted by A. Sanchez *et al*. from their low-cost, low-power underwater acoustic modem [31].

### 4.1. Evaluation of EDETA-e Protocol Underwater

We test the EDETA-e for UWSNs by means of simulation using the ns-3 simulator. For modeling the underwater communication, we use the Underwater Acoustic Networks (UAN) with Thorp as the acoustic propagation model. A set of EDETA-e simulations was performed to tune parameters and to study if the protocol is suitable for this new transmission medium.

#### 4.1.1. Simulation and Scenario Parameters

The protocol was tested in three different deployment areas. The first one with 100 *×* 100 *m*^2^, the second one with 150 *×* 150 *m*^2^ and the last one with 200 *×* 200 *m*^2^. In all of them 100 and 200 nodes where randomly deployed, which gave 6 different scenarios to test. Each scenario was simulated several times and the mean value of all data was calculated. All the simulations were seeded using the number 1290674301 and each repetition was done advancing the run number [5].

In these experiments, the transmission speed was set to 500 bps. This speed could be increased, but we wanted to account for the speed reduction given by the use of CDMA spreading codes.

Since the protocol used was EDETA-e, the number of CHs and their placement was chosen *ad-hoc* for each scenario. Only one sink was deployed at the centre of every scenario. The configuration phase of the EDETA-e protocol was set to last 18,000 s and the nodes were configured to send their data periodically every 250 s. Each node woke up at its defined time interval and sent one byte with its collected data to its CH. After that, each CH had to aggregate these data and send it to its parent in the tree structure until the data reached the sink.

#### 4.1.2. Simulation Results

Table 1 shows the difference in network lifetime for each scenario. A confidence interval in which the specified average values will reside is given with a confidence level of 95%. The average value of the network lifetime for each scenario is fairly stable, varying at most between *±*1.01%. Also a decrease of the network lifetime can be observed with the increase of the deployment’s area, although we believe that this decrease is more strongly related to the existing node density.

**Figure 3.**
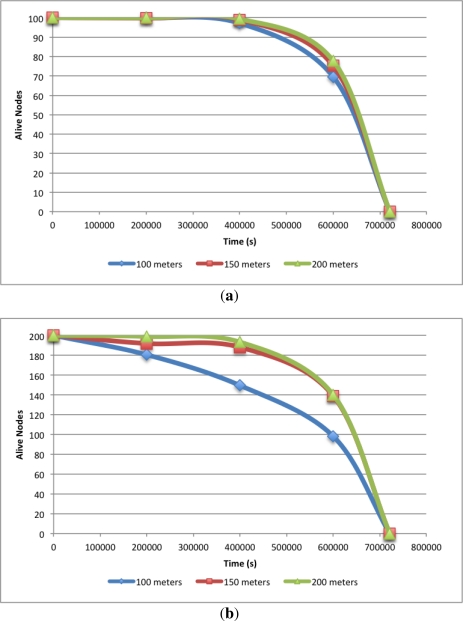
Alive nodes *vs.* time with 100 nodes and 200 nodes. **(a)** 100 nodes scenarios; **(b)** 200 nodes scenarios.

This can be better observed in Figure 3, where a plot of alive nodes over time for the six different scenarios is illustrated. Figure 3(a) depicts these values for the 100 nodes scenarios showing no significant differences between them.

Figure 3(b) shows the evolution of the alive nodes over time for the 200 nodes scenarios and it provides more interesting data to analyse. Although the simulation end time for the three different deployment areas is almost the same, the evolution of the alive nodes over time is different. It can be seen that, at the 100 *×* 100 *m*^2^ deployment area, there are some nodes that deplete their energy almost at the beginning of the simulation and as we increase the scenario area the nodes start to die later. This behaviour is explained by the node density. When node density is high in the deployment area, some nodes will remain awake longer at the configuration phase. This leads to higher energy consumption. Hence, these nodes will die sooner than expected.

**Figure 4.**
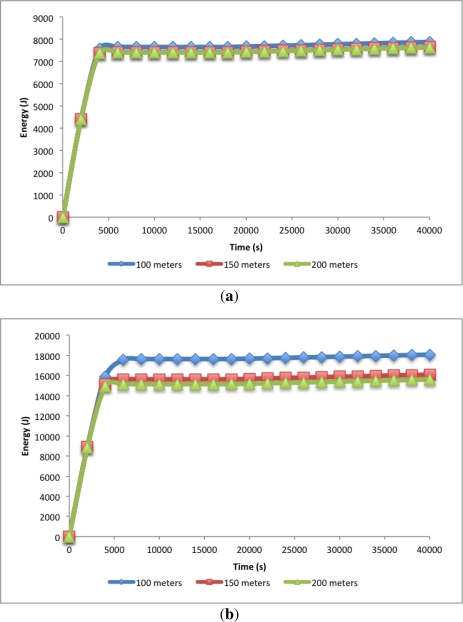
Energy spent *vs.* time with 100 nodes and 200 nodes. **(a)** 100 nodes scenarios; **(b)** 200 nodes scenarios.

The optimum node density will vary in respect to the transmission range. Further studies have to be conducted in order to give more insights in the effect of this parameter. Furthermore, the increase of the transmission range will lead to higher propagation delays and will likely increase the collision probability during the Figure 4 depicts the accumulated spent energy by all nodes over time for the first 40,000 s. Figure 4(a) shows this spent energy for the 100 nodes scenarios. As expected from Figure 3(a) there are no big differences between scenarios. There is one big slope, however, from the beginning of the simulation to 4,000 s. Here the energy consumption increases quite fast. This part corresponds to the first sub-phase of the initialization phase where normal nodes are trying to join a CH, so there is high activity in the network. After this first sub-phase is done and the nodes have joined their cluster, only the CHs will remain awake building the tree schedule to send their data to the sink. So, there is really low energy consumption until the end of the initialization phase. After the initialization phase, the normal operation phase begins. In this scenarios, it corresponds to the period beginning at 18,000 s until the end of the simulation.

In Figure 4(b) the same analysis was done for the 200 nodes scenarios. It depicts differences in the accumulated energy spent by the nodes depending on the area of the scenario, corroborating the results presented by Figure 3(b). As it can be seen, the increase in energy consumption is given in the initialization phase and after that, during the normal operation phase, the slope is the same for all the given scenarios. This indicates that the node density is a determining factor at the initialization phase but after that, it does not have a big influence.

This increase in the energy spent given by the node density comes with an increase of packet collisions at the initialization phase. So, different MACs, in addition to the ALOHA protocol used in this simulations, should be studied to reduce the packet collision at this phase in the underwater channel.

It has to be mentioned that once the network structure was built and the normal operation began, there were no more collisions, giving 100% data packet delivery rate to the sink. So, no data packet was lost during the simulations. configuration phase.

**Figure 5.**
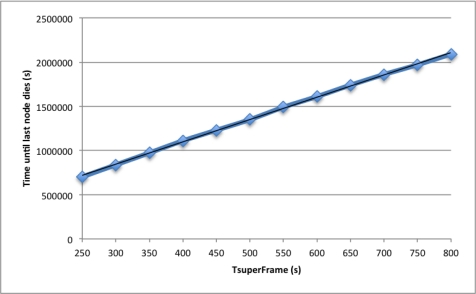
Effect of the *T_SuperFrame_* on the network life time.

Finally, in Figure 5 we study the effect of the *T_SuperFrame_* parameter on the network lifetime. To this end, we performed simulation increasing value of the *T_SuperFrame_* parameter from 250 s to 800 s. As expected, network lifetime grows linearly with this parameter. We calculated a linear regression and obtained the expression noted by Equation (3) with *R*^2^ = 0.999,

(3) $$y=2523.5T_{\mathrm{SuperFrame}}+88841$$

where *R*^2^ is the correlation coefficient and *y* is the estimated network lifetime. This predictable energy consumption allows us to accurately anticipate the network lifetime in absence of errors. For simplicity, only simulation results of 100 nodes and 100 *×* 100 *m*^2^ deployment area are shown in this graph since results for the other five scenario behave in the same way.

### 4.2. Impact Analysis of Different Scheduling and Retransmission Techniques on EDETA-e Protocol

As has been stated before, UWSN suffer from high propagation delays, which can make the traditional TDMA and CSMA medium access techniques inefficient [3]. Scheduling the transmissions allows to avoid collisions and can reduce the propagation delay. However, it is also necessary to introduce an extra time in the schedule to allow the retransmission of the packets in case packet errors occur.

As mentioned in Section 3, nodes using EDETA and EDETA-e employ a TDMA schedule during the operational phase in order to send their data. Each destination node will send an acknowledgement back to the sender when a packet is correctly received.

To evaluate the impact of different scheduling and retransmission techniques on the proposed underwater routing protocol, we will consider two different scheduling techniques, (i) the original TDMA schedule of EDETA and (ii) a delay-aware schedule. In order to perform the last one, the CH has to know the delay to its nodes. After that, the CH schedules the packets using the simplified set of schedule constrains proposed by van Kleunen *et al*. in [17].

We consider three different retransmission techniques. In the first one, a backup transmission is scheduled right after the primary transmission and the node will only use it if there is a packet loss. In the second one a backup transmission is scheduled right after the primary transmission but, in this case, no ACK is sent back to the sender. Therefore, the sender always uses the backup transmission. Finally, in the third one, the transmissions are scheduled only once, however, a CSMA period is scheduled for the nodes to send their data again if there is a packet loss.

In what follows, we introduce different combinations of the scheduling and retransmission techniques used to replace the simple TDMA scheduling and retransmission technique of EDETA.

**TAck** is a TDMA schedule with acknowledgement and data packet loss. A TDMA schedule is used by the nodes to send their data and an ACK is sent back when the data is correctly received. Since this schedule is not delay-aware, the slots have to include the maximum propagation time. Each transmission is scheduled two times to provide a backup slot in case of a data packet error occurs.

**TnoAck** is a TDMA schedule without acknowledgement. A TDMA schedule is used by the nodes to send their data but no ACK is sent back. Hence, the TDMA slot will last for the time needed for the packet transmission and the signal to propagate to the maximum distance.

**DAck** is a delay-aware schedule with acknowledgement and data packet loss. A delay-aware schedule is used by the nodes to send their data and an ACK is sent back when the data is correctly received. Each transmission is scheduled two times to provide a backup slot in case of a data packet error occurs.

**DnoAck** is a delay-aware schedule without acknowledgement. A delay-aware schedule is used by the nodes to send their data but no ACK is sent back. Each packet is scheduled to arrive at the destination right after the previous one.

**DnoAck2** is a delay-aware schedule without Acknowledgement. A delay-aware schedule is used by the nodes to send their data but no ACK is sent back. As explained in the previous section, each packet is scheduled to arrive at the destination right after the previous one but each packet is scheduled and transmitted twice.

**DCsma** is a delay-aware schedule with acknowledgement and CSMA retransmission period. A delay-aware schedule is used by the nodes to send their data and an ACK is sent back when the data is correctly received. If the ACK is not received because of data packet loss, the node will wait until the CSMA period starts in order to send its data again.

#### 4.2.1. Performance Evaluation

In this section, impacts of the scheduling and retransmission techniques introduced in the previous section on EDETA-e are evaluated in order to compare them with the original TDMA approach in terms of delay, packet loss, energy consumption, and number of duplicate packets.

EDETA-e with different scheduling and retransmission techniques was tested in three different deployment areas, *i.e.*, (i) 100 *×* 100 *m*^2^; (ii) 150 *×* 150 *m*^2^; and (iii) 200 *×* 200 *m*^2^. In all of these deployments, 50, 100, and 200 nodes were randomly distributed, which result in 9 different scenarios to test. Each scenario has been simulated several times in order to achieve a confidence interval of *±*3% with a confidence level of 95%. All simulations were seeded using the number 1310572618 and each repetition was done advancing the run number [5].

The configuration phase of the EDETA-e protocol was set to last for 18,000 s and the nodes were configured to send their data periodically every 250 s. Each node woke up at its defined time interval and sent one byte with its collected data to its CH. After that, each CH had to aggregate these data and sent it to its parent in the tree structure until the data reached the sink.

##### TnoAck *vs.* DnoAck

In these experiments, the performance of a TDMA schedule and a delay-aware schedule, both of them without acknowledgement, were evaluated.

**Figure 6.**
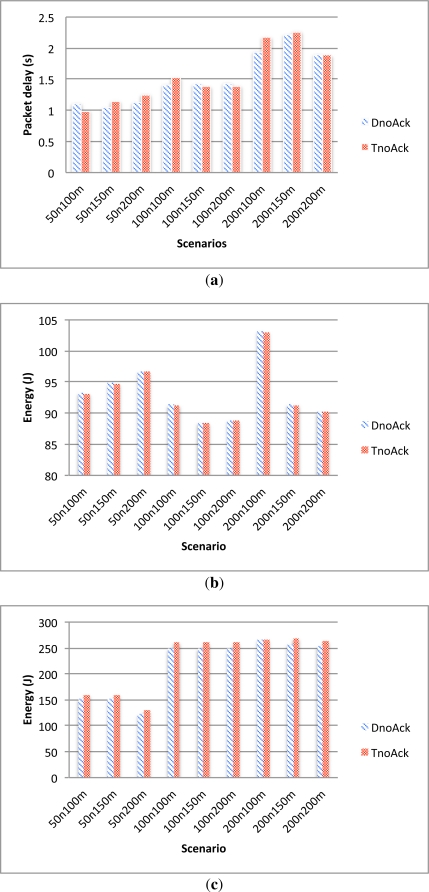
TnoAck and DnoAck comparison. **(a)** Delay; **(b)** Leaf nodes energy consumption; **(c)** CH nodes energy consumption.

Given the hierarchical nature of the protocol, one notes that the packet delay is heavily influenced by the network topology and since the transmission range used in the simulations is relatively small (100 m), the propagation delay is not the dominant factor of the packet delay in these simulations. As it can be seen from Figure 6(a), there are almost no significant differences in packet delay between the two alternatives.

Also, as depicted in Figure 6(b), energy consumption of the leaf nodes is the same for the two cases since, in both alternatives, the nodes only wake up to send their data and then go back to sleep without waiting for any acknowledgement. On the other hand, Figure 6(c) shows that CHs consume less energy when they use the delay-aware schedule compared with when they use the TDMA schedule. This difference in energy consumption is produced at the initialization phase where, given the different scheduling algorithms, the initialization phase is slightly different in both alternatives.

##### TAck *vs.* DAck

In these experiments, the performance of a TDMA schedule and a delay-aware schedule, both of them with acknowledgement and a scheduled backup transmission are evaluated.

**Figure 7.**
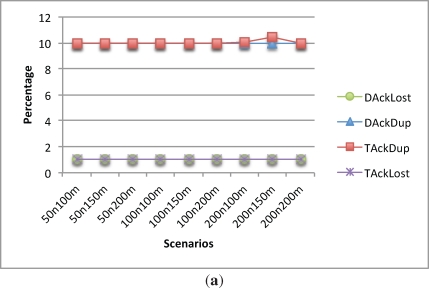

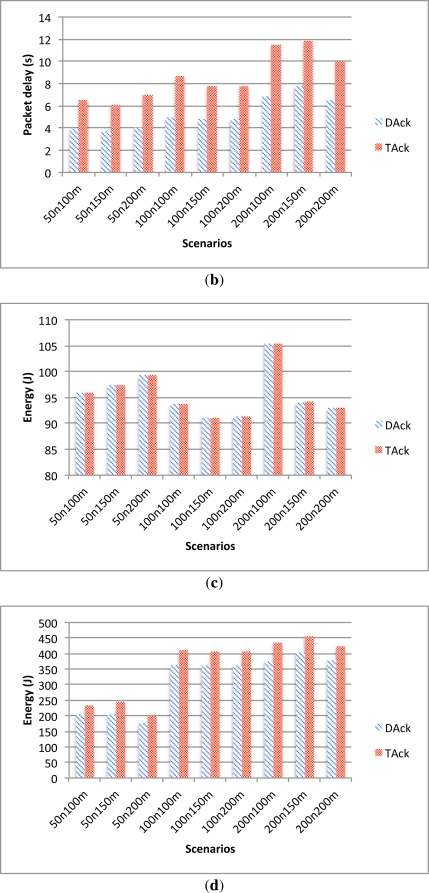
TAck and DAck comparison. **(a)** Lost and duplicate data; **(b)** Delay; **(c)** Leaf nodes energy consumption; **(d)** CH nodes energy consumption.

It can be seen from Figure 7(a) that the behavior of duplicate and lost packets are as expected. Since there is always a backup transmission scheduled right after the main transmission and the data packet error rate was set to be 10%, the packet lost rate is 1% in every scenario and the duplicate data packets is around 10%.

Focusing on the delays, when acknowledgements and poll messages are introduced, the TDMA slots increase. On the other hand, the delay-aware schedule can optimize the data transmissions and by doing so, as depicted in Figure 7(b), it can reduce the delays.

This reduction in the time spent for communication comes with a reduction on the energy consumption of the CHs. Figure 7(d) shows the energy consumption of the CHs during the first 100,000 s of simulation. As it can be seen, the delay-aware schedule consumes less energy than the TDMA schedule.

On the other hand, leaf nodes do not get any benefit from the delay-aware scheduling, as they consume the same amount of energy in every scenario as depicted in Figure 7(c). This happens because the leaf nodes have to wait for the acknowledgement packet and in both cases it is immediately sent after the data packet arrives.

##### CSMA comparison

In these experiments, instead of scheduling a backup data transmission, a CSMA period is scheduled right after the nodes send their data. This allows the nodes that have not received an ACK to send their data again.

**Figure 8.**
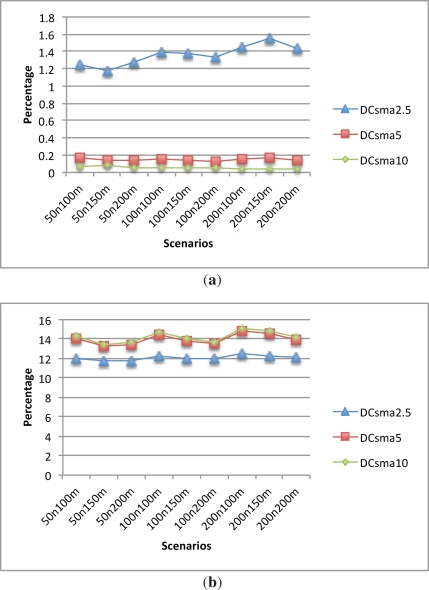

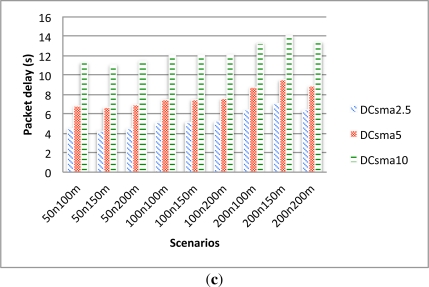
CSMA comparison. Lost, Dup and Data delay. **(a)** Lost data; **(b)** Dup data; **(c)** Delay.

Figure 8(a) shows how lost data packet ratio behaves given the different CSMA period lengths. With a CSMA period of 2.5 s, the packet lost ratio stays between 1.2% and 1.6%. However, when the CSMA time increases to 5 s, the packet lost ratio drops to less than 0.2%. Finally, increasing the CSMA period to 10 s does not offer much benefit, as it decreases the packet lost ratio to less than 0.1%.

Figure 8(b) depicts the percentage of duplicate packets. As expected, the 2.5 s CSMA period has less duplicate data packets than the other alternatives, since there are less retransmissions given the small time scheduled for them. This lesser number of retransmissions also lead to a larger packet lost ratio as explained before. For the 5 and 10 s CSMA period, the percentage of duplicate data packets remains almost the same in all scenarios.

One notes that the packet delay is heavily influenced by the CSMA period length. As can be seen in Figure 8(c), the larger the period length the bigger the delay is.

**Figure 9.**
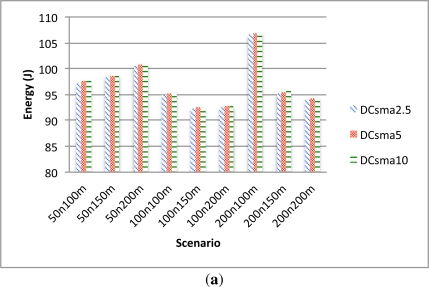

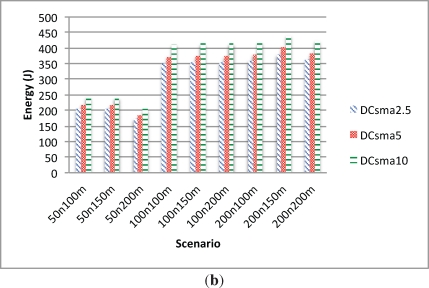
CSMA energy comparison. **(a)** Leaf nodes energy consumption; **(b)** CH nodes energy consumption.

The energy consumption of the CHs is also heavily influenced by the CSMA period length as shown in Figure 9(b). Although a CH changes to a low power consumption mode when it has received all the data from its nodes, the fact that there are more retransmissions produces lower packet lost but increases the energy consumption.

Energy consumption of the normal nodes is also influenced by the CSMA period length although not as heavily as the CHs energy consumption. Figure 9(a) shows that in the scenarios with a 2.5 CSMA period there is less energy consumption than the other ones and there are not significant differences between 5 and 10 s scenarios.

##### DAck *vs.* DCsma2.5 *vs.* DnoAck2

In this section, we compare the two previously studied alternatives, *i.e.*, (i) delay-aware schedule with acknowledgement and delay-aware schedule with a 2.5 s CSMA retransmission period; and (ii) delay-aware schedule with two scheduled transmissions and no acknowledgement (DnoAck2).

**Figure 10.**
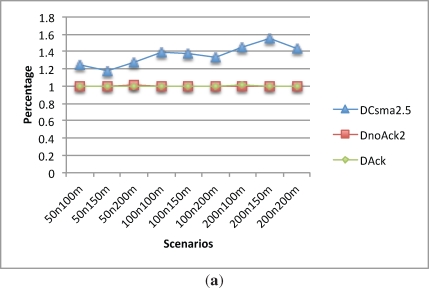

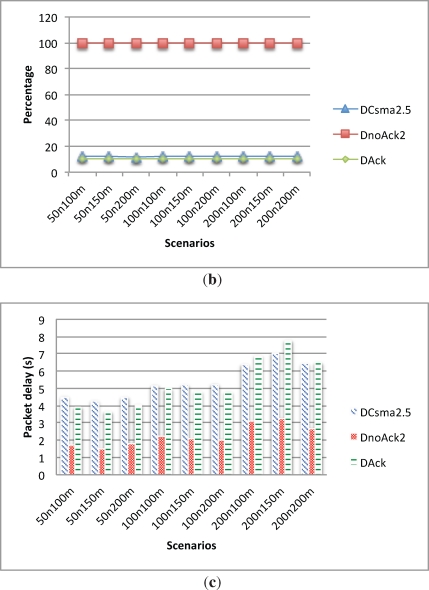
DAck *vs.* DCsma2.5 *vs.* DnoAck2. Lost, Dup and Data delay. **(a)** Lost data; **(b)** Dup data; **(c)** Delay.

In terms of packet lost ratio, duplicate packets and packet delay, the DnoAck2 alternative behaves as shown in Figure 10. The lost packet ratio is the same as the DAck alternative since in both cases the data will be lost if both scheduled data transmission fail. However, DnoAck2 has a 100% duplicate data since each packet is sent twice. On the other hand, the delay of the DnoAck2 alternative is the smallest one of the three protocols.

**Figure 11.**
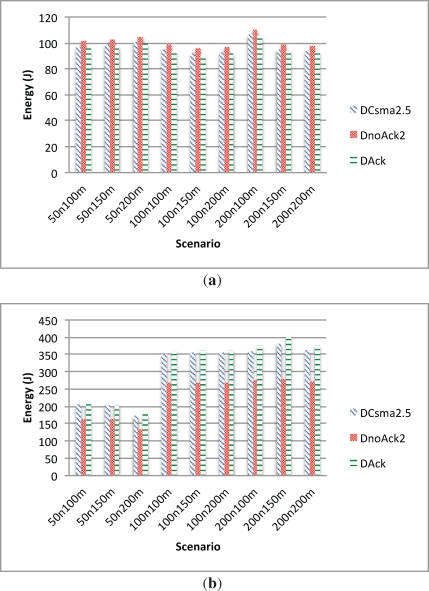
DAck *vs.* DCsma2.5 *vs.* DnoAck2 energy comparison. **(a)** Leaf nodes energy consumption; **(b)** CH nodes energy consumption.

In terms of energy consumption of the leaf nodes (Figure 11(a)), the DnoAck2 alternative has the biggest overhead since it transmits each packet twice. On the other hand, as shown in Figure 11(b), CH nodes using DnoAck2 have the lowest energy consumption. These nodes also have to transmit their data twice, but this extra energy consumption is compensated by the energy saving from not sending acknowledgements back, which also allows to make the communication faster. Hence, CH nodes can remain in low power state during more time.

### 4.3. EDETA-e vs. DBR

In this section, performance of EDETA-e using different scheduling and retransmission parameters will be compared with the performance of DBR using different values for the *δ* and threshold parameters. The *δ* parameter specifies the holding time, and the threshold parameter specifies the minimum depth difference for a packet to be retransmitted.

We simulate DBR with two different sets of parameters: the first one with *δ* = 0.25 and the threshold value of 40 m which according to our tests gives the best energy savings, and the second one with *δ* = 100 and the threshold value of 0 m which gives the best performance in terms of packet delivery ratio.

Both protocols use the same MAC layer (ALOHA protocol) and the same modem parameters, although DBR transmission speed is increased to 1,000 bps since this protocol does not need the usage of CDMA spreading codes. In both protocols, each node sends one byte of data plus its corresponding header every 250 s.

Each scenario has been simulated several times in order to achieve a confidence interval of *±*3% with a confidence level of 95%. All simulations were seeded using the number 1310572618 and each repetition was done advancing the run number [5].

**Figure 12.**
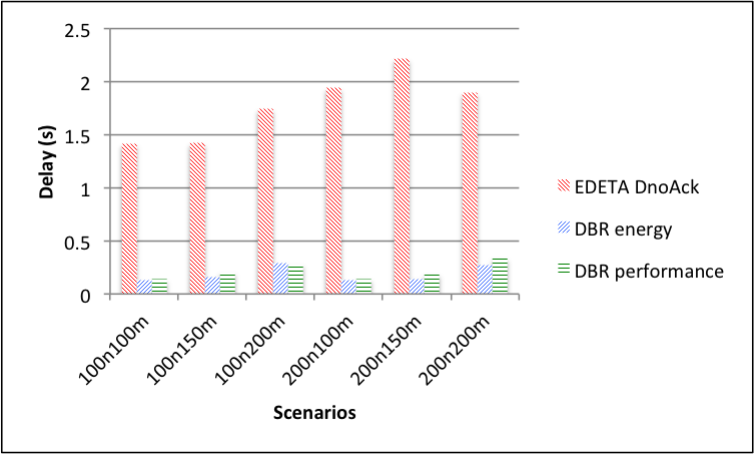
End-to-end delay.

Figure 12 shows the average end-to-end delay for EDETA-e with the delay aware schedule and without acknowledgement. It also shows the parameters set for energy saving and packet delivery ratio performance of DBR. As it can be seen, the average end-to-end delay for EDETA-e is higher than the delay for the two types of DBR.

**Figure 13.**
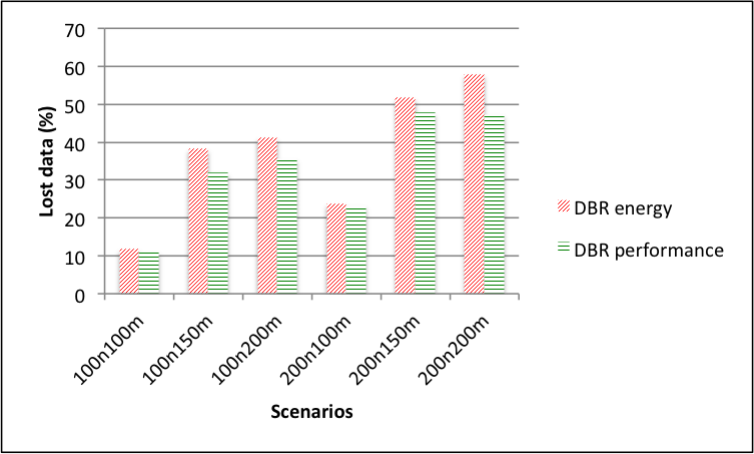
DBR lost data ratio.

However, in terms of lost data due to collisions, EDETA-e experiences no collisions since it is a fully scheduled algorithm. DBR, on the other hand, presents high packet loss as depicted by Figure 13.

The packet loss ratio of DBR in these simulations is higher than the one presented in the original paper because in the original paper authors use a CSMA-like MAC with backoff mechanism which increases the packet delay and indirectly modifies the packet holding time that is a key parameter of the protocol.

**Figure 14.**
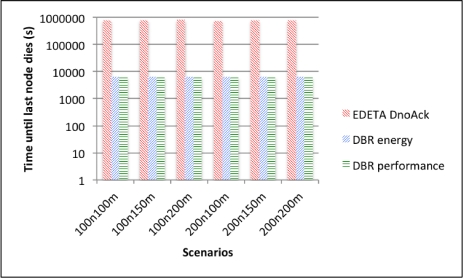
Simulation end time.

Finally the simulation end time for the three alternatives is shown in Figure 14 using a logarithmic scale. As it can be seen, there are no significant differences between the two DBR alternatives. This is because nodes using DBR never use a low-power radio mode. On the other hand, using EDETA-e the last node dies more than 100 times later.

## 5. Conclusions and Future Work

This paper presents a novel routing protocol for underwater wireless sensor networks. EDETA-e is a power-aware routing protocol which minimizes the energy consumption. It organizes nodes in clusters and uses low-power modes at the times in which nodes have no need to be awake. In addition, the protocol adds fault tolerant mechanisms and has time-constrained properties.

We analyze the behavior of EDETA-e protocol in the subaquatic medium by means of simulations in ns-3. The results show high reliability in terms of no data packet loss due to collisions and an optimal energy management during the normal operation phase, allowing the nodes to remain in a low-power state when they have no data to deliver to the sink. Moreover, different scheduling and retransmission techniques applied to a EDETA-e have been simulated and their performance in terms of energy consumption, delays, packet lost rate and duplicate packets has been analyzed.

Results show that, taking advantage of the transmission delay when performing the scheduling can significantly reduce the energy consumption and delays, maintaining the same packet delivery ratio when packet errors are introduced.

The performance evaluation of EDETA-e protocol shows a stable and efficient behavior of clusters and tree structures. It has proven to be very energy efficient compared with the DBR protocol. EDETA-e totally avoids packet loss due to collisions by using ALOHA, the simplest MAC available. Moreover, paths can be dynamically adapted to topology changes and node failures, offering the maximum energy saving without exact location information.

As a consequence of these studies and results, it can be concluded that EDETA-e is a very suitable protocol for subaquatic sensor networks, presenting, in addition, new desired features not present in other approaches in this field.

As future work there are different aspects to deal with. First of all, due to the satisfactory results obtained during the experimentation phase, the proposed protocol is nowadays being implemented in real nodes in collaboration with WSNVAL S.L., a company dedicated to the development of wireless sensor networks both terrestrial and underwater. Thus, we will be able to test different underwater MAC protocols with EDETA in a real underwater environment.

On the other hand, the integration of mobile nodes in the underwater EDETA architecture constitutes another goal. The research in the underwater sensor networks increasingly focuses on mobile networks. In this line we are extending EDETA to support mobility, keeping its properties such as energy-efficiency, scalability, flexibility and reliability.

Finally, we will study the integration of artificial intelligence techniques in order to confer the protocol a greater adaptability in changing surroundings, which will increase even more the applicability of EDETA.

## Figures and Tables

**Table 1.** Network alive time.

**Meters**	**100 × 100**		**150 × 150**		**200 × 200**	
Nodes	100	200	100	200	100	200
Average	702,400 s	704,000 s	693,250 s	689,450 s	700,550 s	693,900 s
Confidence interval	±0.78%	±0.36%	±0.45%	±0.53%	±1.01%	±0.28%
